# Vitamin D deficiency decreases survival of bacterial meningoencephalitis in mice

**DOI:** 10.1186/s12974-014-0208-1

**Published:** 2015-01-07

**Authors:** Marija Djukic, Nadine Sostmann, Thomas Bertsch, Marianne Mecke, Stefan Nessler, Anja Manig, Uwe-Karsten Hanisch, Jakob Triebel, L Cornelius Bollheimer, Cornel Sieber, Roland Nau

**Affiliations:** Department of Geriatrics, Evangelisches Krankenhaus Göttingen-Weende, Göttingen, Germany; Department of Neuropathology, University Medical School Göttingen, Göttingen, Germany; Institute of Clinical Chemistry, Laboratory Medicine and Transfusion Medicine, Paracelsus Medical University, Nuremberg, Germany; Institute for Biomedicine of Aging (IBA), Friedrich-Alexander Universität Erlangen-Nürnberg, Nuremberg, Germany; Hospital of the Order of St. John of God, Regensburg, Regensburg, Germany

**Keywords:** CNS infection, *Escherichia coli*, vitamin D

## Abstract

**Background:**

Meningoencephalitis caused by *Escherichia coli* is associated with high rates of mortality and risk of neurological sequelae in newborns and infants and in older or immunocompromised adults. A high prevalence of neurological disorders has been observed in geriatric populations at risk of hypovitaminosis D.

**Methods:**

*In vivo*, we studied the effects of vitamin D3 on survival and the host’s immune response in experimental bacterial meningoencephalitis in mice after intracerebral *E. coli* infection. To produce different systemic vitamin D3 concentrations, mice received a low, standard, or high dietary vitamin D3 supplementation. Bacterial titers in blood, spleen, and brain homogenates were determined. Leukocyte infiltration was assessed by histological scores, and tissue cytokine or chemokine concentrations were measured.

**Results:**

Mice fed a diet with low vitamin D3 concentration died earlier than control animals after intracerebral infection. Vitamin D deficiency did not inhibit leukocyte recruitment into the subarachnoid space and did not lead to an increased density of bacteria in blood, spleen, or brain homogenates. The release of proinflammatory interleukin (IL)-6 was decreased and the release of anti-inflammatory IL-10 was increased in mice fed a diet with high vitamin D3 supplementation.

**Conclusion:**

Our observations suggest a detrimental role of vitamin D deficiency in bacterial central nervous system infections. Vitamin D may exert immune regulatory functions.

## Background

Death in the acute phase of the disease and neurological as well as neuropsychological sequelae are frequent complications of bacterial central nervous system (CNS) infections. *Escherichia coli* is a Gram-negative bacillus causing local infections in the urinary tract, abdomen, and lungs. Systemic spread of these infections is frequent, leading to sepsis and meningoencephalitis, and is associated with high rates of mortality and morbidity in newborn infants, and in immunocompromised or elderly persons despite advances in antimicrobial chemotherapy [1]. The presence of the capsule K1 confers invasiveness to the strains and enables them to penetrate the blood–brain barrier *in vivo* [2,3]. Several studies in human beings and in the newborn rat model of hematogenous *E. coli* meningoencephalitis suggest that a high degree of bacteremia is required for meningeal invasion [4,5]. The ability of bacteria to achieve high bacterial concentrations in blood, increasing the probability of invasion of the CNS, is greater in immunocompromised individuals (for example, neonates) than in immunocompetent adults, thus explaining the differences in the occurrence of *E. coli* K1 meningoencephalitis [6-8]. Invasion of brain microvascular endothelial cells appears to be a prerequisite for *E. coli* K1 to induce meningoencephalitis [8]. Some *E. coli* K1 structures, such as outer membrane protein A (OmpA), Ibe proteins, and cytotoxic necrotizing factor 1, are necessary for successful bacterial traversal across the blood–brain barrier [8]. In recent years, a significant increase in multidrug-resistant *E. coli* strains has been observed [9]. In Europe, antimicrobial resistance in Gram-negative bacteria is spreading, particularly in *E. coli*, which constitutes a large portion of invasive Gram-negative isolates in European countries [10]. Antibiotics are essential for the control and treatment of *E. coli* infections in human beings and animals. However, it is generally accepted that antimicrobial resistance is associated with the quantity of antibiotic consumption [11]. Vaccination may be an important primary prevention strategy in human beings against most *E. coli* strains. To date, no effective vaccine is available for the prevention of these infections [10]. Therefore, the development of novel therapeutic strategies for these infectious diseases is of high priority. Vitamin D has long been known to play a role in building the skeletal system and in calcium homeostasis; vitamin D deficiency is known to be a cause of rickets and osteomalacia and aggravates osteoporosis [12]. In addition to this well-known role in mineral and skeletal homeostasis, 1,25-dihydroxyvitamin D3 (1,25(OH)_2_D3) affects both innate and adaptive immune responses [13,14]. Cells of the immune system possess vitamin D receptors and are capable of metabolizing the active form of vitamin D (1,25(OH)_2_D3) [15], suggesting vitamin D as an important factor in the immune response to infection [16]. Studies of the innate immune response to pathogens such as *Mycobacterium tuberculosis* have shown that pathogen-recognition receptor (PRR)-mediated activation of localized vitamin D metabolism and signaling is a key event associated with resistance to infection [17]. Epidemiological studies have established that vitamin D deficiency plays an important role in susceptibility to tuberculosis [18]. Vitamin D supplementation showed a beneficial modulating effect on sepsis [19] and on endotoxin shock in mice [20]. We have previously shown in microglia cultures that vitamin D3 deficiency may impair the resistance of the brain against bacterial infections [21]. Taken together, these data indicate an important role of vitamin D3 in the clearance of infections and containment of inflammation by the body’s immune cells. Here we extended our analysis to the action of vitamin D3 *in vivo*. The aims of this study were (i) to investigate the immunomodulatory capacity of vitamin D and (ii) to examine the impact of vitamin D3 supplementation as a preventive or adjuvant therapeutic intervention on the course and mortality of experimental *E. coli* meningoencephalitis.

## Methods

### Vitamin D3 concentration in the blood

To produce different vitamin D3 concentrations in the circulation, mice were fed with a diet containing either low (L-VitD; vitamin D3 concentration below the detection level; normal calcium and phosphate concentrations), standard (S-VitD; vitamin D3 concentration 1,500 IU/kg food; normal calcium and phosphate concentrations), or high (H-VitD; vitamin D3 concentration 75,000 IU/kg food; normal calcium and phosphate concentrations) vitamin D3 concentrations (all from ssniff Spezialdiäten GmbH, Soest, Germany). After 6 weeks, the 25-hydroxyvitamin D3 serum concentrations were measured in the mice using liquid chromatography-tandem mass spectrometry. Serum samples were obtained by puncture of the retroorbital plexus and measured using a MassChrom® 25-OH-Vitamin D3/D2 LC-MS/MS kit (Chromsystems, Munich, Germany). Measurements were made using an AB Sciex API 4000 LC/MS/MS system (AB Sciex, Darmstadt, Germany). The HPLC component was from Shimadzu (Duisburg, Germany). The assay was adapted to a 50 μl sample volume.

### Animals

The animal experiments were approved by the Animal Care Committee of the University Hospital of Göttingen and by the Niedersächsische Landesamt für Verbraucherschutz und Lebensmittelsicherheit, Braunschweig, Lower Saxony, Germany. C57Bl/6 wild-type mice (2 to 3 months old, weight 20 to 30 g, Charles River Laboratory) were used in all experiments [10]. Water and food were available *ad libitum*.

### Bacteria

The *E. coli* strain K1 (serotype O18:K1:H7), originally isolated from the cerebrospinal fluid of a child with neonatal meningoencephalitis (and the gift of Dr. Gregor Zysk, Institute of Medical Microbiology, Düsseldorf, Germany) was used in all experimental infections. Bacteria were grown overnight on blood agar plates, harvested in 0.9% saline and stored at −80 °C. Frozen aliquots were thawed immediately before the experiments and diluted with saline to the required bacterial concentration.

### Induction of meningoencephalitis

For survival experiments, meningoencephalitis was induced by the slow injection of 4,000 colony-forming units (CFUs) of *E. coli* K1 in 10 μl sterile saline (0.9% NaCl) into the right frontal lobe of the cerebral cortex using a 27-gauge disposable needle [22] under intraperitoneal anesthesia with ketamine (100 mg/kg of body weight) and xylazine (10 mg/kg of body weight). All animals resumed their normal behavior after awaking from anesthesia. During the acute disease phase, animals were weighed and scored every 12 h (0, no apparent behavioral abnormality; 1, moderate lethargy; 2, severe lethargy; 3, unable to walk; 4, dead) [19]. Mice with a clinical score of 3 were killed for ethical reasons. In survival experiments, animals were monitored for 14 days after infection. In bacteriological studies, using a 27-gauge needle, 10 μl of a suspension containing 9,000 CFUs of *E. coli* K1 or an equal amount of saline were slowly injected into the right frontal lobe of the cerebral cortex. Mice were killed 20 h after infection.

### Sample processing

Mice that were killed 20 h after infection were anesthetized with ketamine (100 mg/kg of body weight) and xylazine (10 mg/kg of body weight). Blood was drawn by cardiac puncture and 10 μl was used for the determination of bacterial concentrations. The remaining blood was stored at 4°C for 30 min and then centrifuged at 3,000 *g* for 10 min at 4°C. Serum was then transferred to another tube and stored at −20°C. The whole brain and spleen were removed. The cerebellum was dissected from the brain stem. Half of the spleen and the whole cerebellum were homogenized in 0.9% saline. Bacterial titers in homogenates and blood were determined by plating serial 10-fold dilutions in 0.9% saline on sheep blood agar plates (detection limit: 100 CFU/ml, respectively). The whole cerebrum and the other half of the spleen were fixed in 4% paraformaldehyde and then embedded in paraffin. In all experiments, a control group of five mice per group was injected with 0.9% NaCl.

### Histological analysis

Paraffin-embedded, 2 μm coronal brain sections from killed or dead mice from the survival experiments as well as from animals killed 20 h after infection in the bacteriological studies were analyzed. Chloroacetate esterase staining was performed to evaluate the degree of inflammation in three superficial meningeal regions and the hippocampal fissure. This stain is used to detect neutrophils but it can also stain some monocytes or macrophages [23]. The numbers of chloroacetate-esterase-stained leukocytes were counted in one high-power field (×40 objective) per region by a blinded investigator. For each animal, the leukocyte numbers of the individual fields were added and then divided by the number of counted regions.

### Flow cytometry

Anesthetized animals were perfused transcardially with PBS 20 h after infection, and the whole brain was removed and processed. Brain was digested and homogenized with collagenase D (2.5 mg/ml, Roche Diagnostics GmbH, Mannheim, Germany) and DNase I (2 mg/ml, Roche Diagnostics GmbH) using a gentleMACS dissociator (Miltenyi Biotec, Germany). The resultant homogenates were mechanically dissociated and passed through a 70-μm nylon cell strainer (BD Biosciences, Franklin Lakes, NJ, USA). Leukocytes were separated in a 37/70% Percoll gradient (GE Healthcare, Chalfont St Giles, Buckinghamshire, UK). Single cells were stained with the following antibodies: CD45 (30-F11), CD4 (RM4-5), CD27 (LG.3A10), CD11b (M1/70), and Ly6C (HK1.4) purchased from BioLegend (San Diego, CA, USA), CD3 (145-2C11), CD25 (PC61.5), CD19 (eBio1D3) NK1.1 (PK136), and FoxP3 (FJK-16 s) provided by eBioscience (San Diego, CA, USA), and Ly6G (1A8, BD Pharmigen, Franklin Lakes, NJ, USA) and CCR2 (FAB5538A, R&D Systems, Minneapolis, MN, USA). At least 50,000 events were acquired on a FACSCanto II cell analyzer (BD Biosciences) and analyzed using FlowJo software (version 8.8; Tree Star).

### Cytokine and chemokine measurement

Cytokines and chemokines were measured in cerebellum homogenates of mice killed 20 h after infection and in the cerebellum and spleen of dead mice from survival experiments as well as in the cerebellum and spleen homogenates of five mice per group injected with 0.9% sterile saline. Concentrations of IL-6, IL-10, KC (CXCL1), IFN-γ, and MIP-2 (CXCL2) were determined using DuoSet ELISA development kits (R&D Systems, Wiesbaden, Germany). Procedures were performed according to the manufacturer’s instructions. The sensitivity of the assays for these cytokines and chemokines was 7.5 pg/ml.

### Statistics

Vitamin D3 serum concentrations, the weight of animals, bacterial loads in cerebellum and spleen, and the mean number of leukocytes per area are shown as mean and standard deviation (SD) and were analyzed by one-way-ANOVA and corrected for repeated testing with the Bonferroni multiple comparisons test. The cytokine and chemokine concentrations are reported as medians and corresponding interquartile ranges (Q25 and Q75) and were analyzed by the Kruskal-Wallis test and corrected for repeated testing using the Bonferroni method. For survival analysis, the log-rank test based on a Kaplan-Meier plot was used. The clinical scores were reported as box and whiskers (min to max) and were analyzed using the Kruskal-Wallis test. *P* values were corrected for repeated testing with the Bonferroni method. For all analyses, GraphPad Prism version 5 (GraphPad Software, San Diego, CA) was used; *P* ≤ 0.05 was considered statistically significant.

## Results

### Vitamin D3 serum concentrations depended on dietary vitamin D3 content

The analysis of the vitamin D3 metabolite 25-OH-D3 in serum samples obtained after six weeks of experimental diet revealed that the vitamin D3 content in the diet determined the vitamin D serum concentrations of the mice. After 6 weeks of feeding, a diet containing low vitamin D concentrations (L-VitD) resulted in serum 25-OH vitamin D3 concentrations significantly lower than those in S-VitD or H-VitD diet-fed mice (mean ± SD: 5.9 ± 2.6 versus 30.4 ± 4.0 and 172.4 ± 26.1 ng/ml, *P* < 0.05 for all group comparisons).

### Vitamin D3 deficiency led to higher mortality of *E. coli* meningoencephalitis

**Figure 1:**
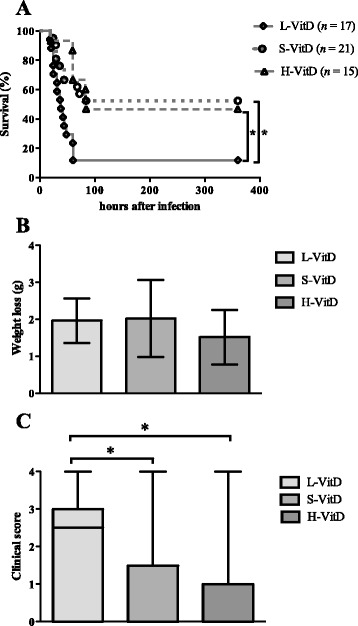
**Adequate vitamin D3 supply decreases susceptibility to** ***E. coli*** **meningoencephalitis. (A)** Survival, **(B)** weight loss, and **(C)** clinical score of mice fed diets containing different amounts of vitamin D3 (low (L-VitD), standard (S-VitD) or high (H-VitD) vitamin D supplementation). Meningoencephalitis was induced by injection of 4,000 CFUs of *E. coli* K1 into the right frontal lobe of the cerebral cortex. Kaplan-Meier curves were compared by log-rank test. Differences in weight loss 24 h after infection are shown as mean and standard deviation (SD) and were analyzed by one-way-ANOVA and corrected for repeated testing with the Bonferroni multiple comparisons test. Clinical scores at 24 h after infection were analyzed using the Kruskal-Wallis test and corrected for repeated testing by the Bonferroni method. Data are shown as box and whiskers with minimum, median and maximum. *P < 0.05.

After intracerebral infection with 4,000 CFUs, 15 of 17 vitamin D-deficient mice (L-VitD), 10 of 21 mice fed a S-VitD diet, and 8 of 15 mice fed a H-VitD diet died (*P* = 0.002 L-VitD versus H-VitD, *P* = 0.003 L-VitD versus S-VitD, log-rank test, Figure 1A). At 20 hours after inoculation with 4,000 CFUs of *E. coli,* all infected animals were slightly lethargic but able to walk and feed. At 24 hours after infection, vitamin D-deficient mice had a tendency towards a greater weight loss (*P* = 0.22; Figure 1B), the difference failing to reach statistical significance because of high interindividual variation. At this time, vitamin D-deficient mice (L-VitD) had higher clinical scores than the two other groups (*P < 0.05; Kruskal-Wallis test, correction for repeated testing with the Bonferroni method, Figure 1C). Between animals fed a S-VitD diet (vitamin D3 concentration 1,500 IU/kg) and animals fed a H-VitD diet (vitamin D3 concentration 75,000 IU/kg), no statistically significant differences concerning weight and clinical score were found (*P* = 0.6, Kruskal-Wallis test).

### Vitamin D3 administration did not influence bacterial titers in CNS and spleen at the end stage of the disease or 20 hours after inoculation with *E. coli*

**Figure 2:**
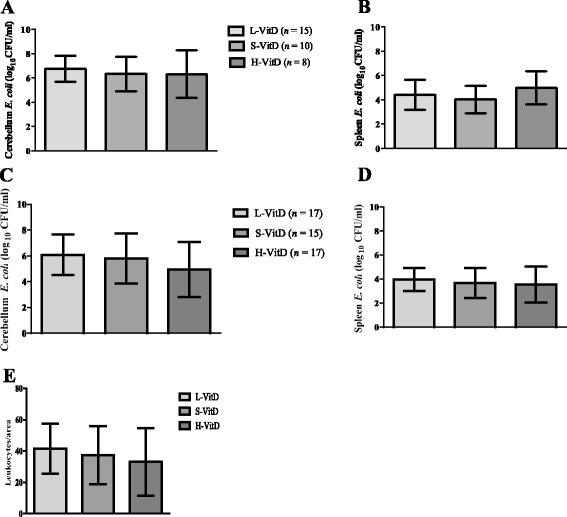
**Vitamin D3 pre-treatment did not modulate bacterial loads and did not influence meningeal inflammation during meningoencephalitis.** Bacterial concentrations in **(A)** the cerebellum and **(B)** the spleen at the end stage of infection in animals that were killed or died during the acute phase of infection and **(C)** in the cerebellum, and **(D)** the spleen 20 h after intracerebral *E. coli* K1 infection. **(E)** The mean numbers of chloroacetate-esterase-stained leukocytes per area in brain sections of mice fed a diet containing different amounts of vitamin D (low (L-VitD), standard (S-VitD) or high (H-VitD) vitamin D supplementation), which were killed after 20 h of *E. coli* meningoencephalitis (*n* ≥ 15). Bars indicate mean and standard deviation (SD). Statistical analysis was performed by one-way ANOVA followed by Bonferroni correction for multiple comparisons. (A,B) *n* ≥ 8, (C,D,E) *n* ≥15 mice per group.

In 1986, Rook and colleagues [17] found in cultured human monocytes that active vitamin D [1,25(OH)2D3], can inhibit the growth of *Mycobacterium tuberculosis*. Therefore, we asked whether vitamin D3 plays any antibacterial role during *E. coli* infection. The densities of viable bacteria in the cerebellum and spleen of animals dying after an infection with 4,000 CFUs of *E. coli* were not different between the three groups (Figure 2A: *P* = 0.8; Figure 2B: *P* = 0.2; one-way ANOVA followed by Bonferroni multiple comparisons test for repeated testing; mean ± SD).

At 20 hours after infection with 9,000 CFU of *E. coli*, the differences of the bacterial titers in the cerebellum (Figure 2C) and spleen (Figure 2D) of mice of all three groups were not statistically significant (Figure 2C, *P* = 0.2; Figure 2D, *P* = 0.6; one-way ANOVA followed by Bonferroni multiple comparisons test for repeated testing). Bacterial titers in the blood of mice of all three groups were also not significantly different. Control mice injected with sterile saline all had sterile cerebellar and spleen homogenates and blood cultures.

### Higher vitamin D3 concentration led to a better clearance of bacteria in mice that survived the infection

After 14 days of infection all surviving mice (*n* = 2) fed a L-VitD diet showed positive bacterial cultures in cerebellum homogenates compared with 20% (*n* = 2/10) of the animals fed a S-VitD diet (*P* = 0.09; Fisher’s exact test). All surviving mice (*n* = 7) fed a H-VitD diet had negative bacterial cultures (100%) in cerebellum homogenates (*P* = 0.03 compared with vitamin D3-deficient mice and *P* = 0.5 compared with mice fed a S-VitD diet; Fisher’s exact test) indicating that high vitamin D3 concentrations positively influence the clearance of bacteria in the CNS.

### Diets with different amounts of vitamin D3 did not affect meningeal inflammation or the recruitment of myeloid cells into the inflamed CNS 20 hours after *E. coli* infection

Infiltrates by granulocytes and monocytes can already be observed in the meninges during bacterial meningoencephalitis at 12 h after infection with *S. pneumoniae* [24]. To investigate the role of vitamin D3 on the meningeal infiltration during *E. coli* meningoencephalitis, we quantified the infiltrates in an early stage of the disease (20 h after infection) by chloroacetate esterase staining. Histological examination of the brains revealed leukocyte recruitment into the subarachnoid space 20 hours after *E. coli* infection in all groups (*n* = 17 per group, Figure 2E). Animals fed a L-VitD diet showed a tendency towards a higher mean number of leukocytes per area compared to animals fed a H-VitD diet, but this difference failed to reach statistical significance (mean ± SD: 41.6 ± 16 versus 33.1 ± 21.4; *P* = 0.4, one-way ANOVA followed by Bonferroni multiple comparisons test for repeated testing). In addition to the histological analysis, we analyzed distinct cell types in the whole brains of mice at 20 h after *E. coli* infection by flow cytometry. The relative numbers of the different cell subtypes among all CD45^+^ cells found in the CNS are shown in Table 1. There were no differences between the groups receiving different amounts of vitamin D3 (*P* > 0.05).

**Table 1:** **Subpopulations of T cells in animals fed a diet containing different amounts of vitamin D 20 hours after infection**

	**Percentage of all CD45** ^**+**^ **cells** ^**a**^		
	**S-VitD**	**L-VitD**	**H-VitD**
T cells (CD45^+^CD3^+^)	11.25 (4/23.53)	7.5 (1.1/18.50)	10.70 (2.75/23.35)
Regulatory T cells (CD45^+^CD4^+^CD3^+^CD25^+^FoxP3^+^)	0.25 (0.04/0.93)	0.05 (0.005/0.17)	0.09 (0.015/0.5)
NK cells (CD45^+^NK1.1CD3^−^)	2.25 (1.525/3.43)	3 (0.95/4.4)	1.3 (1.15/1.65)
Inflammatory monocytes (CD45^+^CD11b^+^Ly6C^high^CCR2^+^)	28.90 (26.95/34.75)	36.20 (25.9/40.85)	27 (18.10/31.20)
Monocytes (CD45^+^CD11b^+^Ly6C^int^CCR2^−^)	35.35 (33.3/43.33)	42.20 (31.4/47.3)	34.40 (25.75/37.85)
Granulocytes (CD45^+^CD11b^+^Ly6G^+^CCR2^−^)	43.25 (28.85/51.58)	26.20 (23/49.95)	34.20 (28.60/54.40)

^a^Data are medians (25th/75th percentiles) determined by forward and side-scatter properties from the whole brain of animals (four or five mice per group) of at least three separate experiments. Significant differences were not found (*P* > 0.05; one-way ANOVA followed by Bonferroni correction for multiple comparisons). L-VitD low vitamin D supplementation; S-VitD, standard vitamin D supplementation; H-VitD, high vitamin D supplementation.

### High vitamin D3 concentrations inhibited the production of proinflammatory cytokine IL-6 and increased production of anti-inflammatory cytokine IL-10, while IFN-γ production in the CNS stayed unaffected

**Figure 3:**
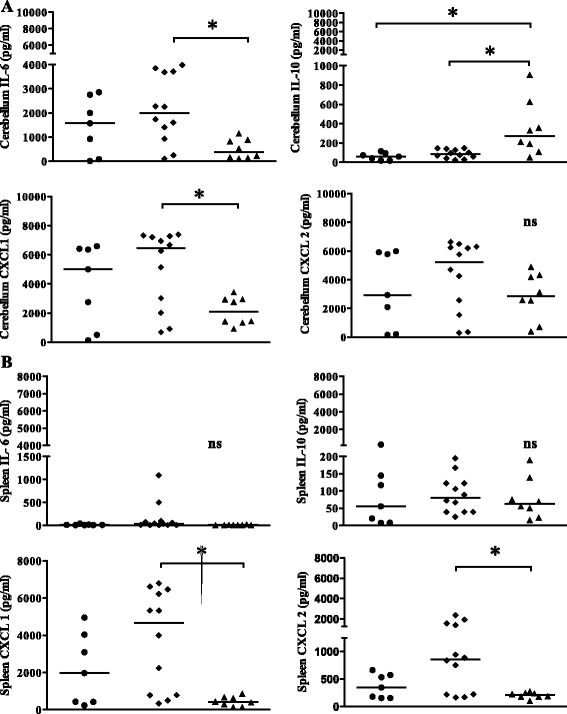
**The protective effect of vitamin D3 correlated with low concentrations of proinflammatory cytokine IL-6 and high concentrations of anti-inflammatory cytokine IL-10 in cerebellum homogenates of mice dying during the acute phase of infection.** Cytokines and chemokines were measured in **(A)** cerebellum and **(B)** spleen homogenates of mice of the survival experiments (time of death or killed because of severe clinical symptoms after intracerebral *E. coli* K1 infection (4,000 CFU/mouse): median = 44 h). Each symbol represents the measurement from an individual mouse (*n* ≥ 7 per group). Horizontal bars indicate median values. Statistical analysis was performed using the Kruskal-Wallis test and corrected for repeated testing with the Bonferroni method (*P < 0.05, ns: not significant). The time of the tissue collection was not the same for all animals. Filled circles, standard vitamin D (S-VitD) diet-fed mice (vitamin D3 concentration 1,500 IU/kg food); filled squares, low vitamin D (L-VitD) diet-fed mice (vitamin D3 concentration less than the detection level); filled triangles, high vitamin D (H-VitD) diet-fed mice (vitamin D3 concentration 75,000 IU/kg food).

**Figure 4:**
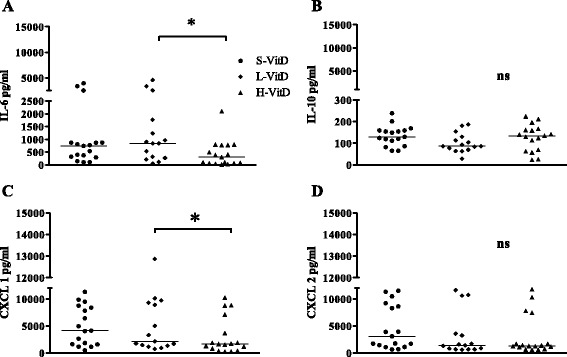
**Pre-treatment with vitamin D3 significantly decreased IL-6 and CXCL 1 in cerebellum homogenates at early infection. (A)** IL-6, **(B)** IL-10, **(C)** CXCL 1 (KC), and **(D)** CXCL 2 (MIP-2) were measured 20 h after intracerebral *E. coli* K1 infection (9,000 CFU/mouse). Each symbol represents an individual mouse (*n* = 17 per group). Horizontal bars indicate median values. Statistical analysis was performed using the Kruskal-Wallis test and corrected for repeated testing with the Bonferroni method (*P < 0.05, ns: not significant). Filled circles, standard vitamin D (S-VitD) diet-fed mice (vitamin D3 concentration 1,500 IU/kg food); filled squares, low vitamin D (L-VitD) diet-fed mice (vitamin D3 concentration under detection level); filled triangles, high vitamin D (H-VitD) diet-fed mice (vitamin D3 concentration 75,000 IU/kg food).

Previous investigations revealed that vitamin D plays important roles in signaling in both the adaptive and innate immune response to viral and bacterial infection [16,25]. Vitamin D modulates the production of many cytokines, such as IL-2, IL-4, IL-6, IL-10, and IFN-γ [26-28]. Macrophage inflammatory protein (MIP-2, CXCL2) is produced by immune cells resident in the brain and attracts monocytes and neutrophils from the bloodstream into the cerebrospinal fluid in acute bacterial meningoencephalitis [29]. Based on the importance of neutrophils and monocytes in controlling bacterial burdens during early *E. coli* K1 meningoencephalitis, we measured IL-6 and IL-10 as key cytokines of the innate defense system during infection and the chemokines CXCL1 (KC) and CXCL2 (MIP-2) as key players in the recruitment of neutrophils into the CNS [30,31]. Infected mice showed alterations in the levels of proinflammatory mediators. The concentrations of IL-6 in the cerebellum of animals that died or were killed during the acute phase of the infection (animals fed a L-Vit D diet: *n* = 12; animals fed a S-VitD diet: *n* = 7; animals fed a H-VitD diet: *n* = 8) and of animals killed 20 h after infection (*n* = 17 for all groups) were significantly decreased in mice fed a H-VitD diet compared with animals fed a L-VitD diet (*P* < 0.05; Figures 3A, 4A; difference between L-VitD versus S-VitD not significant). Animals fed a S-VitD diet also showed a tendency towards higher IL-6 concentrations in the cerebellum than animals fed a H-VitD diet, but this difference failed to reach statistical significance (*P* = 0.2).

In contrast with cerebellar IL-6, IL-10 concentrations were significantly increased in mice with high vitamin D3 supplementation that died during the acute phase of the infection compared with the animals that had received low or standard vitamin D3 supplementation (*P < 0.05; Figure 3A). In mice killed 20 h after infection, IL-10 levels in the cerebellum also tended to be higher in mice with high vitamin D3 supply, the difference failing to reach statistical significance (*P* > 0.05; Figure 4B). The release of IL-6 and IL-10 in the spleens of animals dying during the acute phase of infection was similar in all three groups (*P* > 0.05; Figure 3B). Cerebellar levels of CXCL1 (KC) were also significantly decreased in animals dying during the acute phase of the infection and mice killed 20 h after infection when they had received high vitamin D3 supplementation, as compared with animals fed a L-VitD diet (*P < 0.05; Figures 3A, 4C, differences between L-VitD versus S-VitD not significant). The same effect was found in the spleens of animals dying during the acute phase of infection (*P < 0.05; Figure 3B, differences between L-VitD versus S-VitD not significant).

Although the differences of cerebellar concentrations of CXCL2 (MIP2) in animals dying during the acute phase of infection failed to reach statistical significance (Figure 3A), the highest CXCL2 (MIP 2) concentrations were found in the cerebellum of animals fed a L-VitD diet (medians (25th/75th percentiles): 5,237 (1,801/6,287) pg/ml compared with 2,937 (215.9/5,912) pg/ml in animals fed a S-VitD diet and 2,859 (402.9/4,294) pg/ml in animals fed a H-VitD diet; (Figure 3A)). In the spleen, the concentrations of CXCL2 (MIP2) were significantly increased in animals dying during the acute phase of infection fed a L-VitD diet compared with animals fed a H-VitD diet (*P < 0.05; Figure 3B, differences between L-VitD versus S-VitD not significant). The concentrations of all cytokines and chemokines in the spleen 20 hours after infection (IL-6, IL-10, CXCL1, and CXCL2) were not different among all three groups studied. The cytokine and chemokine levels of uninfected animals (*n* = 5 per group) and in cerebellum and spleen homogenates were all lower than the levels in infected animals. In addition to its influence on bacterial clearance, IFN-γ has the potential to exacerbate inflammation and subsequent pathology, primarily through its ability to modulate the functions of neutrophils and monocytes or macrophages [32]. Vitamin D is considered to suppress the IFN-γ-mediated activation of macrophages. At 20 h after infection, low levels of IFN-γ were found in the cerebellum homogenates of all three groups, indicating that neither *E. coli* infection nor the amount of dietary vitamin D3 substantially influenced the IFN-γ cerebellar concentrations. The levels of IFN-γ measured in the spleen homogenates 20 h after infection also were not influenced by the amount of dietary vitamin D3 (*P* = 0.3; Kruskal-Wallis test). The levels of IFN-γ measured in cerebellum and spleen homogenates of animals dying during the acute phase of infection were all less than the limit of quantification.

## Discussion

Here we provide experimental evidence that in bacterial CNS infection an adequate supply of vitamin D3 decreases susceptibility of the brain to *E. coli* infection and reduces mortality. Moreover, vitamin D has anti-inflammatory properties, illustrated in this study by increased anti-inflammatory IL-10 and decreased proinflammatory IL-6 concentrations in brain tissue. Clinical and experimental observations support the hypothesis that vitamin D has an effect on the immune response to infection and the ability to eliminate pathogens after entry into the host. Vitamin D deficiency may therefore be an underlying cause of infectious diseases and immune disorders [14,16,33]. A central feature of many of these non-classical actions of vitamin D is related to the synthesis of active 1,25(OH)_2_D in a cell-specific manner: enzyme 25OHD-1α-hydroxylase (encoded by the gene *CYP27B1*) is expressed by many extrarenal tissues, including the immune system [14,34]. For example, activated human T- and B-cells can convert the inactive intermediate of vitamin D (also known as 25-hydroxyvitamin D or 25(OH)D) to active 1,25(OH)_2_D *in vitro* [35], and this locally produced 1,25(OH)_2_D acts on immune cells in an autocrine or paracrine fashion [36]. The concentration of the biologically active form of vitamin D, 1,25(OH)_2_D, is dependent on the serum vitamin D (25(OH)D) concentration, the substrate for CYP27B1 [13,37].

To assess the role of vitamin D in an *in-vivo* experimental model of bacterial CNS infection, we analyzed the influence of the infection on the survival and immune response of mice fed a diet containing different amounts of vitamin D3 after injection of *E. coli* directly into the right frontal lobe. The mice did not receive antibiotic treatment; that is, this model tests the ability of mice to combat pathogens after they have entered the brain and cerebrospinal fluid. The importance of vitamin D for the resistance to infections has long been appreciated but poorly understood. This has been especially true for tuberculosis. Indeed, prior to the development of specific drugs for the treatment of tuberculosis, getting out of the city into sunlight (and fresh air) was the treatment of choice [16]. In a survey of patients with tuberculosis in London [38], 56% had undetectable 25(OH)D levels, and an additional 20% had detectable levels below 9 ng/ml (22 nM). A randomized double-blind intervention study showed that supplementation with vitamin D may reduce disease burden in patients with frequent respiratory tract infections [33]. *In-vivo* studies in mice and rats also showed beneficial effects of vitamin D in lipopolysaccharide-induced sepsis [20,39], and antenatal vitamin D therapy improved survival in newborn rat pups and enhanced their lung structure after exposure to endotoxin [40]. The influence of vitamin D on the course of CNS infections is not known. We therefore asked whether vitamin D might modulate the course of and survival from infection after bacterial meningoencephalitis and whether vitamin D could potentially be used therapeutically to alleviate CNS pathology. We observed that vitamin D3-deficient mice died earlier and more frequently than mice fed a diet containing standard or high amounts of vitamin D3. The bacterial burdens in mice dying in the acute phase of infection and mice killed at 20 h were similar in all three groups, that is, the antibacterial action of vitamin D3 in our experimental design was less apparent. The high number of vitamin D3-deficient animals with persistent bacteria in the brain compared with animals with an adequate or high vitamin D3 supply, however, indicated that vitamin D3 also supported pathogen elimination in our model. In an early study, Rook and colleagues [17] demonstrated that active vitamin D, (1,25(OH)_2_D), can inhibit the growth of *Mycobacterium tuberculosis in vitro*. At this time, the physiological significance of this finding was unclear. To clarify the activity of vitamin D against *M. tuberculosis*, Liu and colleagues [41] observed that activation of the toll-like receptor TLR1/2 by a lipoprotein extracted from *M. tuberculosis* reduced the viability of intracellular *M. tuberculosis* in human monocytes and macrophages, concomitant with an increased expression of the vitamin D receptors and of CYP27B1 (the enzyme that produces 1,25(OH)_2_D) in these cells. Killing of *M. tuberculosis* occurred only when the serum in which the cells were cultured contained adequate levels of 25(OH)D, the substrate for CYP27B1. Activated vitamin D (1,25(OH)_2_D) bound to the monocyte vitamin D receptors and then was able to act as a transcription factor leading to the induction of cathelicidin, a potent antimicrobial protein [42], and the promotion of phagocytosis and intracellular killing [41,43,44]. We were also able to show that, in cultured microglia cells, vitamin D3 deficiency led to a decreased phagocytosis and intracellular killing rate of *E. coli* [21]. One reason for the mild antimicrobial properties of vitamin D3 in the present *in-vivo* model might be that, unlike the human *cathelicidin* gene, the mouse gene does not contain a vitamin D response element, and is not induced directly by 1,25(OH)_2_D [16]. Presumably, in addition to cathelicidin, other immune mediators are involved in the vitamin D-dependent immune pathways.

In human beings with bacterial meningoencephalitis, and in animal models of this disease, leukocytes, predominantly myelomonocytic cells such as monocytes, macrophages, and neutrophil granulocytes, quickly enter the subarachnoid space in response to local production of cytokines, chemokines, and other chemotactic stimuli [22]. In terms of *S. pneumoniae* and *E. coli*, we previously showed, *in vivo*, that granulocytes are predominantly involved in the restriction of the multiplication of extracellular bacteria [24,30]. Different vitamin D3 concentrations in the present study did not significantly influence the meningeal invasion with myeloid cells during *E. coli* meningoencephalitis; the mean number of chloroacetate-esterase-stained leukocytes or areas and the recruitment of myeloid cells into the inflamed CNS 20 hours after *E. coli* infection, as assessed by flow cytometry, were comparable in all three groups. A study on the immune response to an allergic stimulus in mice *in vivo* suggested that vitamin D supplementation (100 ng 1,25(OH)_2_D injection) given after the initial period of sensitization prevented high levels of eosinophils associated with a reduced local inflammatory response in bronchoalveolar lavage fluid and lung tissue. Constant vitamin D supplementation (100 ng 1,25(OH)_2_D injection every other day during the whole study period), however, did not reduce the entry of eosinophils into the respiratory epithelia [45]; this is in accordance with our observation.

One mechanism of how vitamin D3 might act beneficially in infections, could be an increase in pathogen phagocytosis with adequate levels of vitamin D [21] and the ability of calcitriol to maintain antimicrobial peptide gene expression [13,14]. Other mechanisms could be the regulation of anti- and proinflammatory compounds by vitamin D. For this purpose, we investigated the pattern of cytokine and chemokine production, a central feature in the development of neuroinflammation, neurodegeneration, and demyelination in the CNS [46,47]. In bacterial meningoencephalitis, after the pathogen crossed the blood–brain barrier, microglia can respond directly to intact bacteria or to bacterial cell wall compounds and produce a wide array of inflammatory mediators, including TNF-α, IL-6, IL-12, keratinocyte-derived chemokine (CXCL1/KC), CCL2/MCP-1, CXCL2/MIP-2, and CCL5/RANTES [48,49]. Neuronal damage in bacterial meningoencephalitis is caused by the dual effects of an overwhelming inflammatory response and the direct action of bacterial toxins [50,51].

In this study, *E. coli* led to the production of numerous cyto- and chemokines in the brain and spleen, including IL-6, IL-10, CXCL1 and CXCL2 shortly after bacterial exposure. IL-6 in the cerebellum, CXCL1 in the cerebellum and spleen and CXCL2 in the spleen were clearly down-regulated in high vitamin D3-fed animals, whereas the bacterial concentrations were not influenced. Our data imply that the protective role of vitamin D3 is related to the reduced release of proinflammatory IL-6 and other chemokines, such as CXCL1 and CXCL2. Moreover, anti-inflammatory IL-10 release [52] was significantly increased in mice dying in the acute phase and fed a diet containing a high vitamin D3 concentration compared with mice dying in the acute phase and fed a diet containing low or standard vitamin D concentrations. This is in accordance with previously published data that vitamin D can increase IL-10 production [16,53]. In murine *S. pneumoniae* meningitis, the absence of IL-10 was associated with higher proinflammatory cytokine and chemokine concentrations and more pronounced infiltrates [54]. IL-10 reduced sepsis-associated hippocampal neuronal damage as a result of pneumococcal sepsis in mice overexpressing IL-10 [55]. Also, intravenously administered recombinant IL-10 reduced the level of cerebrospinal fluid pleocytosis, cerebral edema, and intracranial pressure in a rat model of pneumococcal meningoencephalitis [56]. In our study the IFN-γ levels of animals dying in the acute phase in all three groups were below the level of quantification. In early infection (20 h), low, but approximately equal IFN-γ levels were measured, suggesting that IFN-γ was not involved in the protective action of an adequate vitamin D supply.

## Conclusions

Our experiments identified for the first time a detrimental *in-vivo* effect of vitamin D deficiency on survival in experimental *E. coli* meningoencephalitis. A diet containing a standard amount of vitamin D3 (1,500 IU/kg) is sufficient to decrease the susceptibility of the brain against CNS infection. A diet containing a high amount of vitamin D3 (75,000 IU/kg) supported pathogen elimination and can inhibit inflammation in the CNS. Our results emphasize the necessity of an adequate vitamin D supply to prevent CNS infection. High-dose vitamin D may be able to modulate the inflammatory response to CNS infections. Whether high-dose vitamin D has a protective effect in combination with antibiotics remains to be studied.

## Abbreviations

ANOVA: analysis of variance
CFU: colony-forming unit
CNS: central nervous system
ELISA: enzyme-linked immunosorbent assay
HPLC: high-performance liquid chromatography
H-VitD: high vitamin D3 concentration
IFN: interferon
IL: interleukin
L-VitD: low vitamin D3 concentration
OmpA: outer membrane protein A
PRR: pathogen-recognition receptor
SD: standard deviation
S-VitD: standard vitamin D3 concentration
TLR: toll-like receptor

## References

[1] Russo TA, Johnson JR (2000). Proposal for a new inclusive designation for extraintestinal pathogenic isolates of *Escherichia coli*: ExPEC. J Infect Dis.

[2] Silver RP, Aaronson W, Vann WF (1988). The K1 capsular polysaccharide of *Escherichia coli*. Rev Infect Dis.

[3] Xie Y, Kim KJ, Kim KS (2004). Current concepts on *Escherichia coli* K1 translocation of the blood–brain barrier. FEMS Immunol Med Microbiol.

[4] Kim KS, Itabashi H, Gemski P, Sadoff J, Warren RL, Cross AS (1992). The K1 capsule is the critical determinant in the development of *Escherichia coli* meningitis in the rat. J Clin Invest.

[5] Dietzman DE, Fischer GW, Schoenknecht FD (1974). Neonatal *Escherichia coli* septicemia - bacterial counts in blood. J Pediatr.

[6] Briongos-Figuero LS, Morchón-Simón D, Aparicio-Blanco M, Garea García-Malvar MJ (2008). Spontaneous meningitis due to *Escherichia coli* in the adult: a case report. Rev Clin Esp.

[7] Pitt J (1978). K-1 antigen of *Escherichia coli*: epidemiology and serum sensitivity of pathogenic strains. Infect Immun.

[8] Kim KS (2002). Strategy of *Escherichia coli* for crossing the blood–brain barrier. J Infect Dis.

[9] Pouillot F, Chomton M, Blois H, Courroux C, Noelig J, Bidet P, Bingen E, Bonacorsi S (2012). Efficacy of bacteriophage therapy in experimental sepsis and meningitis caused by a clone O25b:H4-ST131 *Escherichia coli* strain producing CTX-M-15. Antimicrob Agents Chemother.

[10] Allocati N, Masulli M, Alexeyev MF, Di Ilio C (2013). *Escherichia coli* in Europe: an overview. Int J Environ Res Public Health.

[11] van Duijn PJ, Dautzenberg MJ, Oostdijk EA (2011). Recent trends in antibiotic resistance in European ICUs. Curr Opin Crit Care.

[12] Schwalfenberg GK (2011). A review of the critical role of vitamin D in the functioning of the immune system and the clinical implications of vitamin D deficiency. Mol Nutr Food Res.

[13] Hewison M (2012). Vitamin D and immune function: an overview. Proc Nutr Soc.

[14] Hewison M (2011). Vitamin D and innate and adaptive immunity. Vitam Horm.

[15] Holick MF (2007). Vitamin D deficiency. N Engl J Med.

[16] Bikle DD (2008). Vitamin D and the immune system: role in protection against bacterial infection. Curr Opin Nephrol Hypertens.

[17] Rook GA, Steele J, Fraher L, Barker S, Karmali R, O'Riordan J, Stanford J (1986). Vitamin D3, gamma interferon, and control of proliferation of *Mycobacterium tuberculosis* by human monocytes. Immunology.

[18] Khoo AL, Chai L, Koenen H, Joosten I, Netea M, van der Ven A (2012). Translating the role of vitamin D3 in infectious diseases. Crit Rev Microbiol.

[19] Møller S, Laigaard F, Olgaard K, Hemmingsen C (2007). Effect of 1,25-dihydroxy-vitamin D3 in experimental sepsis. Int J Med Sci.

[20] Horiuchi H, Nagata I, Komoriya K (1991). Protective effect of vitamin D3 analogues on endotoxin shock in mice. Agents Actions.

[21] Djukic M, Onken ML, Schütze S, Redlich S, Götz A, Hanisch UK, Bertsch T, Ribes S, Hanenberg A, Schneider S, Bollheimer C, Sieber C, Nau R (2014). Vitamin D deficiency reduces the immune response, phagocytosis rate, and intracellular killing rate of microglial cells. Infect Immun.

[22] Nau R, Wellmer A, Soto A, Koch K, Schneider O, Schmidt H, Gerber J, Michel U, Brück W (1999). Rifampin reduces early mortality in experimental *Streptococcus pneumoniae* meningitis. J Infect Dis.

[23] Ysebaert DK, De Greef KE, Vercauteren SR, Ghielli M, Verpooten GA, Eyskens EJ, De Broe ME (2000). Identification and kinetics of leukocytes after severe ischaemia or reperfusion renal injury. Nephrol Dial Transplant.

[24] Mildner A, Djukic M, Garbe D, Wellmer A, Kuziel WA, Mack M, Nau R, Prinz M (2008). Ly-6G^+^CCR2^−^ myeloid cells rather than Ly-6C^high^CCR2^+^ monocytes are required for the control of bacterial infection in the central nervous system. J Immunol.

[25] Beard JA, Bearden A, Striker R (2001). Vitamin D and the anti-viral state. J Clin Virol.

[26] Lemire JM, Adams JS, Kermani-Arab V, Bakke AC, Sakai R, Jordan SC (1985). 1,25-Dihydroxyvitamin D3 suppresses human T helper/inducer lymphocyte activity *in vitro*. J Immunol.

[27] Abbas AK, Murphy KM, Sher A (1996). Functional diversity of helper T lymphocytes. Nature.

[28] Romagnani S (2006). Regulation of the T cell response. Clin Exp Allergy.

[29] Prinz M, Kann O, Draheim HJ, Schumann RR, Kettenmann H, Weber JR, Hanisch UK (1999). Microglial activation by components of gram-positive and -negative bacteria: distinct and common routes to the induction of ion channels and cytokines. J Neuropathol Exp Neurol.

[30] Ribes S, Regen T, Meister T, Tauber SC, Schütze S, Mildner A, Mack M, Hanisch UK, Nau R (2013). Resistance of the brain to *Escherichia coli* K1 infection depends on MyD88 signaling and the contribution of neutrophils and monocytes. Infect Immun.

[31] Hu W, Nessler S, Hemmer B, Eagar TN, Kane LP, Leliveld SR, Müller-Schiffmann A, Gocke AR, Lovett-Racke A, Ben LH, Hussain RZ, Breil A, Elliott JL, Puttaparthi K, Cravens PD, Singh MP, Petsch B, Stitz L, Racke MK, Korth C, Stüve O (2010). Pharmaco-logical prion protein silencing accelerates central nervous system autoimmune disease via T cell receptor signalling. Brain.

[32] Mitchell AJ, Yau B, McQuillan JA, Ball HJ, Too LK, Abtin A, Hertzog P, Leib SL, Jones CA, Gerega SK, Weninger W, Hunt NH (2012). Inflammasome-dependent IFN-γ drives pathogenesis in *Streptococcus pneumonia* meningitis. J Immunol.

[33] Bergman P, Norlin AC, Hansen S, Rekha RS, Agerberth B, Björkhem-Bergman L, Ekström L, Lindh JD, Andersson J (2012). Vitamin D3 supplementation in patients with frequent respiratory tract infections: a randomised and double-blind intervention study. BMJ Open.

[34] Zehnder D, Bland R, Williams MC, McNinch RW, Howie AJ, Stewart PM, Hewison M (2001). Extrarenal expression of 25-hydroxyvitamin D(3)-1a-hydroxylase. J Clin Endocrinol Metab.

[35] Chen S, Sims GP, Chen XX, Gu YY, Chen S, Lipsky PE (2007). Modulatory effects of 1,25-dihydroxyvitamin D3 on human B cell differentiation. J Immunol.

[36] Sundaram ME, Coleman LA (2012). Vitamin D and influenza. Adv Nutr.

[37] Gombart AF, Borregaard N, Koeffler HP (2005). Human *cathelicidin antimicrobial peptide* (*CAMP*) gene is a direct target of the vitamin D receptor and is strongly up-regulated in myeloid cells by 1,25-dihydroxyvitamin D3. FASEB J.

[38] Ustianowski A, Shaffer R, Collin S, Wilkinson RJ, Davidson RN (2005). Prevalence and associations of vitamin D deficiency in foreign-born persons with tuberculosis in London. J Infect.

[39] Asakura H, Aoshima K, Suga Y, Yamazaki M, Morishita E, Saito M, Miyamoto K, Nakao S (2001). Beneficial effect of the active form of vitamin D3 against LPS-induced DIC but not against tissue-factor-induced DIC in rat models. Thromb Haemost.

[40] Mandell E, Seedorf G, Gien J, Abman SH (2014). Vitamin D treatment improves survival and infant lung structure after intra-amniotic endotoxin exposure in rats: potential role for the prevention of bronchopulmonary dysplasia. Am J Physiol Lung Cell Mol Physiol.

[41] Liu PT, Stenger S, Li H, Wenzel L, Tan BH, Krutzik SR, Ochoa MT, Schauber J, Wu K, Meinken C, Kamen DL, Wagner M, Bals R, Steinmeyer A, Zügel U, Gallo RL, Eisenberg D, Hewison M, Hollis BW, Adams JS, Bloom BR, Modlin RL (2006). Toll-like receptor triggering of a vitamin D-mediated human antimicrobial response. Science.

[42] Risso A (2000). Leukocyte antimicrobial peptides: multifunctional effector molecules of innate immunity. J Leukoc Biol.

[43] Zanetti M (2004). Cathelicidins, multifunctional peptides of the innate immunity. J Leukoc Biol.

[44] Gombart AF, Saito T, Koeffler HP (2009). Exaptation of an ancient Alu short interspersed element provides a highly conserved vitamin D-mediated innate immune response in humans and primates. BMC Genomics.

[45] Matheu V, Bäck O, Mondoc E, Issazadeh-Navikas S (2003). Dual effects of vitamin D-induced alteration of Th1/Th2 cytokine expression: enhancing IgE production and decreasing airway eosinophilia in murine allergic airway disease. J Allergy Clin Immunol.

[46] Smith JA, Das A, Ray SK, Banik NL (2012). Role of proinflammatory cytokines released from microglia in neurodegenerative diseases. Brain Res Bull.

[47] Glass CK, Saijo K, Winner B, Marchetto MC, Gage FH (2010). Mechanisms underlying inflammation in neurodegeneration. Cell.

[48] Hanisch UK, Prinz M, Angstwurm K, Häusler KG, Kann O, Kettenmann H, Weber JR (2001). The protein tyrosine kinase inhibitor AG126 prevents the massive microglial cytokine induction by pneumococcal cell walls. Eur J Immunol.

[49] Rock RB, Gekker G, Hu S, Sheng WS, Cheeran M, Lokensgard JR, Peterson PK (2004). Role of microglia in central nervous system infections. Clin Microbiol Rev.

[50] Braun JS, Novak R, Murray PJ, Eischen CM, Susin SA, Kroemer G, Halle A, Weber JR, Tuomanen EI, Cleveland JL (2001). Apoptosis-inducing factor mediates microglial and neuronal apoptosis caused by pneumococcus. J Infect Dis.

[51] Gerber J, Nau R (2010). Mechanisms of injury in bacterial meningitis. Curr Opin Neurol.

[52] Ramesh G, MacLean AG, Philipp MT (2013). Cytokines and chemokines at the crossroads of neuroinflammation, neurodegeneration, and neuropathic pain. Mediators Inflamm.

[53] Yu X, Zhang X, Zhao B, Wang J, Zhu Z, Teng Z, Shao J, Shen J, Gao Y, Yuan Z, Wu F (2011). Intensive cytokine induction in pandemic H1N1 influenza virus infection accompanied by robust production of IL-10 and IL-6. PLoS ONE.

[54] Zwijnenburg PJ, Van der Poll T, Florquin S, Roord JJ, Van Furth AM (2003). Interleukin-10 negatively regulates local cytokine and chemokine production but does not influence antibacterial host defense during murine pneumococcal meningitis. Infect Immun.

[55] Orihuela CJ, Fillon S, Smith-Sielicki SH, El Kasmi KC, Gao G, Soulis K, Patil A, Murray PJ, Tuomanen EI (2006). Cell wall-mediated neuronal damage in early sepsis. Infect Immun.

[56] Koedel U, Bernatowicz A, Frei K, Fontana A, Pfister H-W (1996). Systemically (but not intrathecally) administered IL-10 attenuates pathophysiologic alterations in experimental pneumococcal meningitis. J Immunol.

